# Therapy-induced cardiotoxicity in breast cancer patients: a well-known yet unresolved problem

**DOI:** 10.15190/d.2019.2

**Published:** 2019-03-31

**Authors:** Diana Ruxandra Florescu, Diana Elena Nistor

**Affiliations:** Faculty of Medicine, Carol Davila University of Medicine and Pharmacy, Bucharest, Romania

**Keywords:** Breast cancer, cardiotoxicity, cardio-oncology, anthracyclines, trastuzumab.

## Abstract

Breast cancer is the second most commonly diagnosed cancer, being one of the main health issues that needs to be addressed worldwide. New therapies have led to a remarkable increase in survival rates, which is unfortunately overshadowed by their negative impact on cardiac structure and function in disease-free patients. Since anthracyclines and trastuzumab cause the most undesired outcome in breast cancer patients - cardiac-related mortality, they have been widely studied. However, other therapies (such as hormonal therapy, tyrosine kinase inhibitors, anti-VEGF drugs etc.) can also affect the cardiovascular system and lead to ischemia, hypertension or vascular thromboembolism. Even though excessive research has been conducted in thepast decades, there are still no guidelines regarding the most adequate methods neither to detect and prevent severe cardiotoxicity that can finally lead to heart failure and ultimately death nor for the further management of patients after cardiotoxicity is detected. Biomarkers of ischemia (troponins T and I) and of overload (BNP and NT-proBNP) in association with periodic echocardiographies (assessment of the global longitudinal strain) are two of the most important means used by physicians in the evaluation of cardiac disease in this group of patients. Given that no internationally accepted guidelines for screening and surveillance of different populations exist, the cardio-oncology team is crucial in the management of these patients, their collaboration resulting in individualized treatment regimens. After careful evaluation of different variables (treatment effects, malignancy status, and the patient's pre-existing conditions), a decision is made to either reduce the dosage or rate of administration, change the medication or interrupt the treatment and initiate the cardioprotective therapeutic associations. Consequently, it is an absolute necessity the development of customized treatment guidelines and the conduction of multiple clinical studies in order to demonstrate their effect on long-term survival.

## **SUMMARY**

**1. Introduction**

**2. Definition of cardiotoxicity**

**3. Breast cancer therapy**

*3.1* *Hormonal therapy*

*3.2* *Targeted therapy*

*3.3* *Chemotherapy*

*3.4* *Radiation therapy*

**4. Associated risk factors modulating individual risk for developing treatment-related cardiac dysfunction**

**5. Types of LV dysfunction and their detection**

*5.1 Systolic dysfunction*

*5.2 Diastolic dysfunction*

*5.3 Subclinical dysfunction*

**6. Early detection of cardiac side-effects and follow-up**

**7. How to reduce cardiac side-effects: cardio-protective associations**

**8. Risk-benefit approach: taking the best decision whether treatment should be continued or not for the best outcome**

**9. Conclusion**

## **1. Introduction**

Breast cancer is the second leading cause of cancer mortality in women of all ages after lung cancer, with the highest incidence amongst malignancies in developed countries^1,2^. The increasing incidence led to the development of a variety of treatment options and to an improvement in means of prognosis and cancer-related mortality.

Another major cause of worldwide mortality and morbidity is represented by cardiovascular diseases. Since many of the therapies used to treat cancer can induce cardiac toxicity and cause heart failure even in previously healthy women, those suffering from pre-existing cardiac diseases are more prone to severe outcomes. Unfortunately, many of the theoretically low-risk patients develop cardiotoxicity. However, it is important to assess the risk and to offer prophylaxis where it is necessary^3^. There have been developed risk scores on the basis of age (the European Society of Cardiology guidelines state age, in particular for those over fifty years old, as a risk factor for cardiac toxicity), type of chemotherapy and comorbidities such as hypertension, diabetes mellitus, coronary disease, atrial fibrillation/flutter, and renal failure^4,5^.

The most important factor in choosing the treatment regimen is the immunohistochemical testing used to measure the expression of treatment targeted receptors. Breast cancer is therefore classified, based on these findings, in HR (hormonal receptors) status: ER (estrogen receptors) +/- PR (progesterone receptors) +/-. Depending on the expression of the protein HER2/neu (human epidermal growth factor receptor 2) breast cancer is classified in HER2+ or HER2-. Another type is represented by the triple negative breast cancer (TNBC). While the subtypes of breast carcinoma with receptors positivity can be treated with targeted therapies, the aggressive TNBC must be treated with classic chemotherapy^6^.

According to the 4th European Society of Oncology - European Society for Medical Oncology (ESO-ESMO) International Consensus Guidelines for Advanced Breast Cancer, breast cancer treatment should be guided by HR and HER2 status, previous therapies and the associated toxicity, stage of the disease (locally advanced or metastatic), age, menopausal status (important in terms of endocrine therapy) and comorbidities. Moreover, the decision must respect the patient’s preferences, socio-economic and psychological status and, of course, the availability of the therapy in the patient’s geographical area^7^.

Even though recent advances in oncologic therapy allow patients to live a prolonged life, severe complications such as the different types of therapy-induced toxicity, are still the main cause of death. Cardiac toxicity is the second cause of morbidity and mortality in breast cancer survivors^8^. Given the prolonged life expectancy due to new therapies, it is crucial to diagnose and prevent further cardiac dysfunction (such as left ventricle dysfunction and cardiomyopathy), since early detection can improve the outcome due to existent therapeutic measures^9,10^.

The recognition of preclinical changes and the risk stratification based on preexisting cardiac diseases could result in an uninterrupted or unmodified therapy which is often required due to the life-threatening complications^11^.

These complications, which are not only related to cardiac dysfunction but the entire cardiovascular system, are seen more and more due to the increased women’s survival rates^9,10^.

For early-stage breast cancer, a patient is more likely to die of heart disease than cancer, cardiotoxic chemotherapy being stated in the American College of Cardiology / American Heart Association (ACC/AHA) Staging System for Heart Failure as one of the main five high-risk factors of developing heart failure^12^. Radiation therapy also has great importance in survival after breast cancer. In older women, therapy-associated mortality from heart failure exceeds cancer-related mortality after ten years^5^. One study concluded that after 12 years, the probability of death because of treatment complications and cancer was the same in older women^13^.

## **2. Definition of Cardiotoxicity**

Current vision accepts cardiotoxicity as a decline in left ventricular ejection fraction (LVEF) of at least 10% to a final value of under 53%, in repeated evaluations (echocardiography, cardiac magnetic resonance imaging, and multiple-gated acquisition-MUGA scan)^14^. However, therapy for breast cancer can affect the cardiovascular system in many ways. Complications include arrhythmias, arterial hypertension, myocardial ischemia, thromboembolism, pericarditis etc. There have been defined two types of cardiac toxicity:

Type I: anthracycline-induced, dose-related, irreversible.

Type II: trastuzumab-induced, not related to dose, reversible^10^.

The traditional classification divides cardiotoxicity induced by cancer treatment in type I (anthracycline-induced) and type II (trastuzumab-induced). This simplified classification is no longer accurate since anthracycline and trastuzumab are often used together in breast cancer treatment and their cardiotoxicity is strongly intensified^15^. Moreover, the use of radiotherapy further amplifies the risk of developing left ventricle dysfunction^15^, while the association with other cancer treatments with a potentially harmful effect on the cardiac function makes the classification incomplete.

After using an agent that could potentially determine cardiac toxicity it is important to search for signs and symptoms of heart damage. The most feared complications are cardiomyopathy and left ventricle (LV) dysfunction. The main strategy is to recognize and then closely follow especially those patients that are at high risk for cardiotoxicity, as they may not always be symptomatic. Regular follow-ups (clinical examination and echocardiography) and early initiation of protective therapy can easily prevent otherwise fatal results. Because of the devastating outcome of cardiac lesions, their presence is translated as a lack of surveillance and prophylaxis.

LV dysfunction, although asymptomatic at first, is associated with an increased risk of developing heart failure. It can be reversible (if the patient uses trastuzumab) or it can have a different cause. Early after chemotherapy, LV dysfunction is a marker for cardiotoxicity, but later in the follow-up, the association between medication and heart failure is more difficult since heart failure can have multiple etiologies (hypertension, diabetes mellitus, old age etc.)^3^.

**Table 1** briefly summarizes the mechanisms of cardio-vascular side-effect of cancer therapy.

**Table 1:** Types of chemotherapy, immunotherapy and radiotherapy and their molecular mechanisms of action; adapted from^16^ with permission

Therapy	Anticancerous mechanism	Cardiotoxicity mechanism
Anthracyclines	Inhibit DNA synthesis	Oxidative stress (cardiomyocyte apoptosis) Inhibition of topoisomerase activity (DNA damage)
Alkylating agents	Inhibit DNA synthesis (by DNA alkylation)	Inhibit long-chain fatty acid oxidation
Antimicrotubule agents	Inhibit cell division (repression of polymerization/depolymerization of microtubules)	Activate Neuronal Calcium Sensor 1 (NCS1) causing Ca²⁺ overload
Monoclonal antibodies	Bevacizumab: inhibits angiogenesis (anti VEGF); Trastuzumab: inhibits cell proliferation (anti EGF/HER2/neu receptor)	Oxidative stress (cardiomyocyte apoptosis)
TKIs	Inhibit cell proliferation Determine apoptosis	Mitochondrial impairment Cardiomyocyte apoptosis
Radiation therapy	Damages cell DNA Inhibits cell division Determines apoptosis Inhibits angiogenesis	Causes endothelial dysfunction Induces fibrogenesis and atherosclerosis Oxidative stress

## **3. Breast Cancer Therapy**

### *3.1 Hormonal Therapy*

ER therapies are described as drugs that block estrogen receptors (tamoxifen, toremifene), drugs that lower estrogen levels (aromatase inhibitors-AIs) or ovarian suppression.

#### *3.1.1 Drugs that block estrogen receptors*

Tamoxifen: acts as an anti-estrogen in breast cancerous cells, while other organs see it as an estrogen. Therefore, it is both an antagonist and an agonist, so it is called a Selective Estrogen Receptor Modulator (SERM). It can be used both as adjuvant and neoadjuvant therapy. It is more frequently used for women at a premenopausal age. Tamoxifen can be administered in all stages of breast cancer. It has been shown to reduce plasma levels of low-density lipoprotein cholesterol, which may be a protective factor for cardiovascular events^17^. However, the Early Breast Cancer Trialists’ Collaboration Group conducted a meta-analysis which did not demonstrate a significant reduction in cardiovascular mortality in tamoxifen-treated patients^18,19^.

#### *3.1.2 Drugs that work by lowering estrogen levels*

Aromatase inhibitors (AIs): letrozole, anastrozole, exemestane. This therapy exerts its effect by blocking the conversion of androgens to estrogen, so they reduce the plasma and tissue levels of estrogens^20^. They are usually used after menopause when estrogen is produced in the fat tissue by an enzyme (aromatase) and can also be used in younger women in association with ovarian suppression. They increase levels of cholesterol, however, they are not associated with increased cardiovascular death, but the association of anastrozole has been linked to more frequent episodes of angina and to hypertension^21,22^.

A recent study confirms that tamoxifen increases the risk of venous thromboembolism and observes an increased incidence of vascular diseases in the AIs-treated patients when compared to those treated with tamoxifen, which again questions the cardioprotective use of tamoxifen^23^.

#### *3.1.3 Ovarian suppression*

Ovarian suppression is usually used for pre-menopausal women with early-stage or metastatic breast cancer. Besides oophorectomy, which requires surgical intervention, there are other ways in which ovarian suppression can be performed: LHRH (Luteinizing Hormone-Releasing Hormone) analogs (goserelin, leuprolide): they induce temporary menopause and can be used in monotherapy or in association with other antiestrogenic drugs and chemotherapy drugs which cause dramatic damage in women’s ovaries (can lead to a temporary or permanent loss of function and is sometimes an unintended result of chemotherapy drugs used for breast cancer treatment)^24^.

### *3.2 Targeted Therapy*

#### *3.2.1 Targeted Therapy for HR (+) Breast Cancer*

For many ER(+)/PR(+) patients hormone therapy is helpful, but it can be improved by the addition of targeted therapy, such as cyclin-dependent kinases (CDK) 4/6 inhibitors (palbociclib, ribociclib, abemaciclib)^24^.

#### *3.2.2 Targeted Therapy for HER-2 (+) Breast Cancer*

*Monoclonal Antibodies*: trastuzumab (a humanized monoclonal antibody), pertuzumab, bevacizumab, ado-trastuzumab emtansine.

Trastuzumab (Herceptin) is a humanized anti-HER2 monoclonal antibody used in aggressive cancers with overexpression of human epidermal growth factor receptor 2 (HER2/ERbB2). The proposed mechanism of cardiotoxicity is related to changes in contractile proteins and mitochondria. Despite its well-known cardiac toxicity, it is widely used both in metastatic breast cancer as well as in adjuvant therapy, due to its capacity of dramatically improving survival rates^25^.

Pertuzumab is a monoclonal antibody that has the capacity of blocking HER2 dimerization with other HER2 receptors and it is approved both as neoadjuvant therapy and in the metastatic stages (in association with trastuzumab and docetaxel). A combination of two anti-HER2 drugs has shown not only improved rates of complete responses when used as neoadjuvant therapy, but also an increased survival when used in metastatic cancer, without a relevant increase in cardiotoxicity^19,26^.

Bevacizumab (Avastin), another monoclonal antibody used to treat breast cancer is responsible for arterial hypertension and inhibition of Vascular Endothelial Growth Factor (VEGF) and receptor signaling, which causes endothelial cell injury^11,23^. It is associated with a 5-fold increase in heart failure risk^27^. It can also exacerbate cardiovascular complications in patients with preexisting arterial hypertension^28,29^.

*Kinase Inhibitors (TKI)*: lapatinib, neratinib.

Lapatinib is an oral TKI of HER2 and epidermal growth factor receptor 1 (EGFR1) and it is approved in metastatic breast cancer. Although not common, it may be associated in some cases with LV failure^30^.

#### *3.2.3 Targeted Therapy for* *BRCA (+) Breast Cancer: olaparib and talazoparib*** ^24^

### *3.3 Chemotherapy***

Chemotherapy can be used after surgery (adjuvant chemotherapy), before surgery (neoadjuvant chemotherapy used for locally advanced cancers) or as the main treatment for advanced breast cancer. There are many different types of drugs used to treat breast cancer and they can be used in various combinations, depending on the tumor’s type, level of invasion and patient’s characteristics.

#### *3.3.1 Anthracyclines*

Doxorubicin, epirubicin: although there is well-known cardiotoxicity of doxorubicin (or other anthracyclines), there are no guidelines for the surveillance of breast cancer survivors in terms of cardiac function. Some organizations recommend careful surveillance of these women during and after breast cancer treatment and, in some cases, even the initiation of cardioprotective therapy before the occurrence of symptoms. However, treating patients in an asymptomatic state has two major drawbacks: cost and efficiency (medication to breast cancer survivors may be potentially harmful). Further studies are required in order to assess if the benefits outweigh the risks and the costs of this type of management^31^.

The well-known type I chemotherapy-related cardiac disease (CRDC), anthracyclines-induced, has many underlying mechanisms, such as the formation of reactive oxygen species the impairment of the DNA repair pathways, interaction with the topoisomerase-II-beta enzyme in the myocytes and activation of mitochondrial topoisomerase-I, which leads to Fe²⁺ overload, energy depletion, damages transcription. As a result, the damage is irreversible, with a high probability of recurrent and progressive dysfunction in the attempt of rechallenge^14,19,32^.

Type I CRDC was known to be proportional to the cumulative exposure^14,33^. New evidence indicates that underlying severe cardiac damage may result even at low doses, especially in patients with preexisting cardiovascular comorbidities or risk factors^33,34^. One study found that doxorubicin could induce cardiac toxicity in doses of less than 500 mg/m2 while another one in doses of less than 300mg/m2^33,35^. The most common toxic effects are decreased left ventricular ejection fraction leading to heart failure, arrhythmias, pericarditis and myocarditis^36^.

Epirubicin has been shown to cause arrhythmias in some patients^30^.

#### *3.3.2 Alkylating Agents*

Cyclophosphamide, ifosfamide, melphalan: they act by inhibiting DNA transcription, therefore affecting protein synthesis^37^. Cyclophosphamide and ifosfamide can determine LV dysfunction shortly after initiation of therapy and may be dose-related (≥150 mg/kg and 1.5g/m2, and >12.5 g/m2 respectively)^25,38^. Cyclophosphamide is commonly associated with myopericarditis and arrhythmias^30^.

#### *3.3.3 VEGF inhibitors*

Some monoclonal antibodies (e.g. bevacizumab) and Tyrosine Kinase Inhibitors (TKI): work by inhibiting angiogenesis^39^.

#### *3.3.4 Antimicrotubule Agents*

Paclitaxel, docetaxel: inhibit the disassembly of microtubules, therefore interrupting cell division^40^. They are especially dangerous since they can interfere with the anthracycline excretion, which increases the risk of LV dysfunction (especially if associated with high doses of anthracyclines). Longer intervals between taxanes and anthracyclines could potentially reduce toxicity^41^.

#### *3.3.5 Antimetabolites* (fluorouracil, capecitabine)

Fluorouracil mostly determines myocardial ischemia and arrhythmias, which are often reversible with treatment discontinuation. However, these events can cause an increase in mortality, therefore it is important to diagnose early stages of cardiac damage and to take into consideration that antimetabolites often determine asymptomatic events. Possible risk factors for fluorouracil cardiotoxicity could be cardiac comorbidities, continuous infusion treatment or association with cisplatin^42^. Further studies are required in order to understand the magnitude of antimetabolites' adverse cardiac effects.

### *3.4 Radiation Therapy***

There are two types of radiation therapy that can be used in breast cancer therapy: external beam radiation (more common) and internal radiation, also called brachytherapy. It can be used after surgery or in metastatic stages^24^. Although it reduces the risk of local recurrence, it causes secondary cardiac effects, because of the heart’s presence in the irradiation field^43^.

Radiotherapy-associated cardiac injury occurs as a combination of microvascular and macrovascular effects^36^. The most common cause of cardiac impairment is the consequence of fibrosis, which leads to ventricular dysfunction and restrictive cardiomyopathy, but radiotherapy can also result in heart failure as a result of acute myocarditis^25^. The myocardial infarction rate after radiation is proportional to the mean whole heart dose^44^.

Recently, heart shields and breast boards have proven their utility in reducing heart irradiation during breast cancer radiotherapy treatment. However, their use depends on the patients’ and their tumor’s characteristics: for the use of heart shield the patient needs to have a clearly visible tumor bed, while the breast board only brings benefits for women with breast volume over 750 cm³ (the presence of the heart within the irradiation field is reduced by 87%)^45-47^.

## **4.** **Associated Risk Factors Modulating Individual Risk for Developing Treatment-related Cardiac Dysfunction**

Before initiation of treatment, the patients can be divided into three categories, based on the existence of cardiovascular diseases, age, LVEF before treatment.

**Figure 1:**
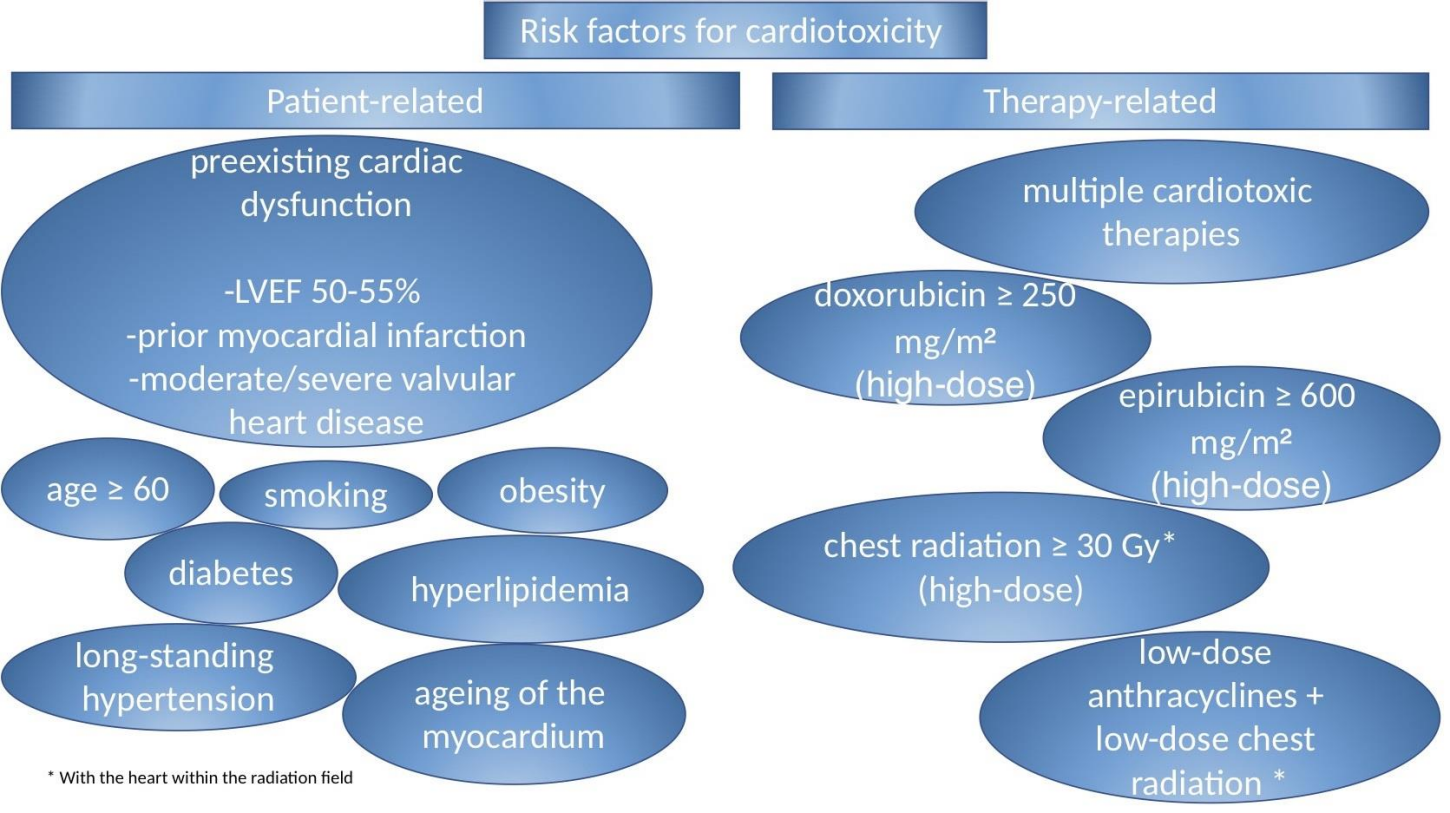


Those at high-risk must not receive anthracycline treatment, while those at low risk will receive normal doses of anthracycline and regular echocardiographic monitorization. Immunotherapy (monoclonal antibodies used in HER+ breast cancer) can also induce severe cardiotoxicity. Factors related to increased risk are previous exposure to anthracyclines, arterial hypertension, low LVEF and age over 65^10,48-50^ (**Figure 1**).

## **5. Types of LV Dysfunctions and their Detection**

### *5.1 Systolic LV Dysfunction*

Besides clinical evaluation, LVEF measured by echocardiography is the first step towards diagnosing systolic dysfunction. This is defined by the American Society of Echocardiography (ASE) and European Association of Cardiovascular Imaging as “a decrease in the LVEF of>10% points, to a value of <53% which should be confirmed by repeated cardiac imaging in 2-3 weeks after the baseline study”^14^. 3D (three-dimensional) echocardiography and the use of contrast agents are strongly recommended for monitoring function of the LV and also quantify the decrease of the LVEF. The latest guidelines recommend that the frequency of LVEF measurement should be determined by the physicians, accordingly to the patient’s characteristics and clinical condition.

Multiple-gated acquisition (MUGA) scan has its limitations considering information on cardiac structure or diastolic function, besides the negative effect of the exposure to ionizing radiation. A previous study also suggests that MUGA scan cannot predict cardiotoxicity in doxorubicin-treated patients experiencing chronic heart failure since it showed no relevant drop of LVEF^51^.

Cardiac MRI (magnetic resonance imaging) is the gold standard for assessing not only the function of the LV but also the cardiac structure. Unfortunately, it cannot always be used, since it may not be available in some regions or because of its cost. Moreover, magnetic resonance is totally dependent on the patient’s tolerance and cooperation, which may sometimes be problematic, especially in cancer patients. Regardless of the imagistic modality, early detection is crucial in order to reduce the dose or the regimen of treatment, with or without the initiation of cardioprotective therapy. As mentioned before, there is no guideline for managing cardiac toxicity in breast cancer treatment, so a multidisciplinary dedicated team must decide the course of action for every individual case^52,53^.

### *5.2 Diastolic LV Dysfunction*

It is strongly recommended to monitor diastolic dysfunction during cancer therapy^25^. At the moment, the hypothesis that diastolic dysfunction occurs prior to the systolic one leads to careful surveillance of tissue relaxation, filling pressures (E/e’) and tissue Doppler indices during echocardiographic evaluation^14,54^.

### *5.3* *Subclinical LV Dysfunction*

#### *5.3.1 Biomarkers*

Their most relevant advantage of biomarkers is that they are minimally invasive and easily reproducible. Troponins (Tn) I and T remain the most used biomarkers in the study of cancer drug cardiotoxicity^25,55^. Studies have shown that persistent TnI increase is associated with a more severe LV dysfunction and an increased incidence of cardiac events (in comparison with only transient increases in troponin levels)^55,56^.

Recently, a new type of troponin has become available: high-sensitive assay, which can determine even low concentrations of troponin. Studies show that high-sensitive troponin I (hs-TnI) can predict cardiomyopathy induced by trastuzumab. This is very important since trastuzumab cardiotoxicity is not related to dose, which makes its effect unpredictable^9^. A study was conducted in order to investigate if the hs-TnT assay is able to detect subclinical myocardial damage anthracycline - or trastuzumab – induced. The results showed that hs-TnT assay may be able to predict cardiotoxicity in breast cancer patients treated with one of these two drugs. The requirements are that periodic measurement is included in medical practice in order to detect and apply a more aggressive prophylactic strategy. However, further studies are required before the introduction of this measurement as a daily medical practice^57^. While BNP (brain natriuretic peptide) and NT-proBNP (N-terminal-proBNP) are more likely to predict heart failure and are associated with increased all-cause mortality, troponins have demonstrated their impact in detecting patients prone to acute myocardial infarction among those at high-risk for coronary artery disease (because of cancer treatment)^58^.

#### *5.3.2* *Echocardiography*

Global longitudinal strain (GLS) is the main tool used in practice in order to detect the early changes in the myocardial contractile function. However, there is no consensus regarding the value that can predict toxicity. The 2016 European recommendation states that a decrease of more than 15% could predict cardiotoxicity, while under 8% excludes its diagnosis, leaving a large grey area between those two values^14,59^. A study conducted on breast cancer women treated with anthracyclines and/or trastuzumab showed a significant drop in the value of LV GLS starting from the third month. The conclusion was that GLS is an independent predictor of cardiac toxicity, with a cut-off of -14%^60^.

## **6. Early Detection of Cardiac Side-effects and Follow-up**

Physicians’ purpose is to detect cardiac toxicity induced by medication in a reversible phase. This phase is also subclinical, which makes regular follow-ups essential in reducing morbidity and mortality. A multidisciplinary team must be involved in the patient’s follow-up. Correlation between biomarkers and cardiac imaging is crucial for the early diagnosis of cardiac dysfunction.

A comparison of the different investigations used to diagnose cardiac disease in cancer patients is presented in **Table 2**.

**Table 2:** Comparison of different investigations used to diagnose cardiac disease in oncology patients; adapted from^61^ with permission.

Investigation	Advantages	Disadvantages
Biomarkers	Widely available Easily reproducible Reduced cost Early detection of cardiac damage (preclinical state)	Lack of specificity Need for dynamic assessment Values could depend on associated comorbidities
Echocardiography	Widely available Ability to measure subtle markers of abnormality e.g. GLS (global longitudinal strain) Assessment of functional implications of coronary artery disease (stress echocardiography) Full assessment of valve disease Full assessment of diastolic function	Inter and intra-observer variability (less with 3D echocardiography or with contrast echocardiography) Difficulty in obtaining optimal images in some patients
CMR (cardiac magnetic resonance)	Accurate and easily reproducibly assessment of the ejection fraction Assessment of functional implications of coronary artery disease (stress CMR) Assessment of cardiac fibrosis which may be related to chemotherapy	Reduced availability Cost Needs patient’s tolerance and cooperation
Nuclear Cardiology	Long-established technique for assessing ejection fraction with significant literature-base	Inability to assess subtle markers of cardiac function Inability to assess valve disease or pericardial effusions Radiation
CTCA (computed tomography of the coronary arteries)	Anatomical assessment of coronary artery disease	Reduced availability Radiation

## **7. How to Reduce Cardiac Side-effects: Cardio-Protective Associations**

Of critical importance is recognizing and if possible, preventing cardiac toxicities in cancer patients. Their requirements for individualized strategies should be performed based on risk stratification and associated complications, which often result in interruption of therapy. Therefore, assessment of preclinical implications and the search for methods to reduce the treatment-related toxicity should be a priority, especially since not only do cancer therapies induce cardio-vascular complications, but also oncology patients have pre-existent heart diseases^62,63^.

While the importance of a multi-disciplinary team consisting of cardiology and oncology specialists is essential for the management of these patients, the prevention of irreversible complications should include a multi-step approach. The first episode towards the desired outcome is the detection of high-risk patients and the modification of their cardiovascular risk factors. Another option is using different strategies for reducing the additional toxicity induced by the therapy, like dose-reduction or avoidance of certain associations (i.e. trastuzumab + anthracyclines), using cardio-protective drugs (beta-blockers, ACE-inhibitors, statins) when cardiac dysfunction has already developed, or ultimately, withholding the therapy^62^.

Therapy-related modifications, such as prolonging infusion time, reducing the volumes with simultaneous administration of furosemide when treatment is administered intravenously and prolonging the time period between infusions for cyclical treatment have the greatest impact on cardiac and vascular function and therapy-induced dysfunctions^64^.

Leading to heart failure, of great importance is the reduction in left ventricle systolic and diastolic functions. One study documented a greater deterioration of LV diastolic function in patients without Lisinopril and Rosuvastatin association compared with the patients in the study group^65^. A double-blind, randomized, placebo-controlled study investigated the effects of carvedilol in patients receiving treatment with anthracyclines, the prophylactic use of this cardioprotective drug resulting in the prevention of LVEF decline at a broad range of carvedilol doses. The importance of this information is that the side-effects of beta-blockers appear to be dose-related^33^. In contrast, another clinical trial concluded that carvedilol had no influence on the onset of LVEF reduction, but it contributed to a significant reduction of diastolic dysfunction and troponin levels^66^. A similar effect on circulating biomarkers (cTnI and cTnT) with a lower increase in their serum concentration was seen in patients treated with metoprolol and candesartan, with the reduction of early myocardial disease, but with the need of further, long-term studies and patient follow-up^67^.

Another beta-blocker, nebivolol, was found to reduce the incidence of anthracyclines-related cardiomyopathy, with the same requirement of long-term results^68^.

A different drug, dexrazoxane, an iron-chelator, is known for the prevention of anthracycline-related cardiac toxicity and acts by preventing the formation of ROS, however with no overall effect on survival. Moreover, there are some concerns about dexrazoxane’s negative involvement in doxorubicin’s antitumor effect, by inhibiting Top2a cleavage complexes in addition to Top2b-DNA cleavage complexes^69^.

Chrysophanol, a natural anthraquinone compound isolated from Rhubarb, has shown a reduction of cardiac apoptosis and mitochondrial injury and cell PARylation levels in vitro but has failed to show any improvement in animal studies or clinical trials. However, further studies may demonstrate their importance^70^.

Different studies and clinical trials report aerobic exercise as having an important impact on individual risk factors, and on reducing the harmful effects of breast cancer medication on the cardiovascular system, by acting on improving the antioxidant capacity and reducing the oxidative stress^71^.

Thus, the beneficial effects of exercise can be summarized in the improvement of cardiorespiratory function, immunity, rates of treatment completion, patient physical strength, and reduction of side-effects^64^.

While under treatment, echocardiography follow-up and biomarkers assessment are recommended. If these reveal pathological findings, such as abnormal levels of biomarkers, reduced LVEF (<50%) or abnormal GLS (global longitudinal strain), it can be recommended to start treatment with ACE inhibitor/ angiotensin 1 blocker or beta-blocker therapy^58,62,72^.

## **8. Risk-Benefit Approach: Taking the Best Decision Whether Treatment Should Be Continued or Not for the Best Outcome**

The burden of a life-saving decision relays on a multidisciplinary team consisting of an oncologist, cardiologist and radiation therapy specialist^73,74^. There are still some unanswered questions:

*Which cardiac function markers should be used while monitoring oncology patients? When precisely to start the cardio-protective therapy? Which therapy would be the most efficient and how to monitor its efficacy?^61^*

Despite not having a commonly accepted answer, the cardio-oncology-radiology collaborative model’s decision should always be based on certain factors which can be grouped into three categories. The first category consists of the most relevant aspects to be taken into consideration – detection and monitoring of the cardiac dysfunction. These indicate, in correlation with patient’s characteristics and decision, cancer type and stage (second category) and treatment regimen used (third category), if medication should be continued or not, with or without cardio-protective associations^74,75^.

**Figure 2:**
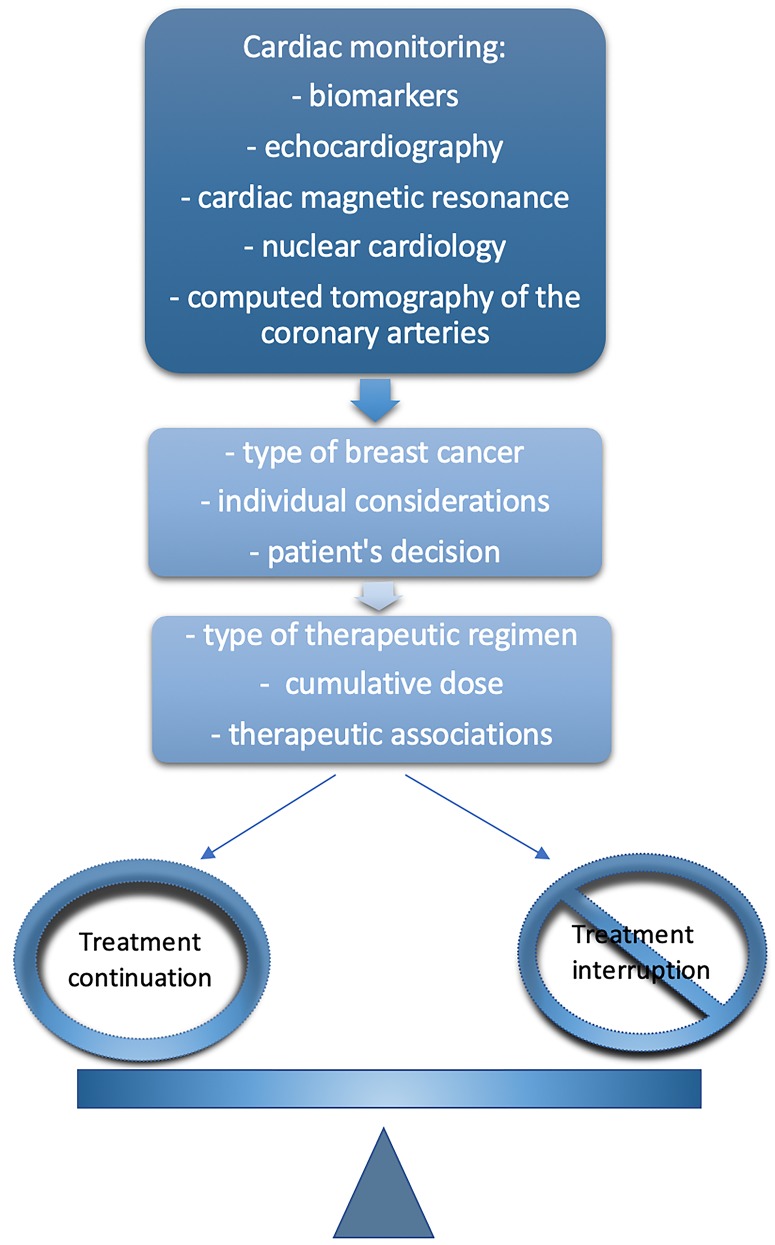
Risk-benefit approach to continuing or interrupting therapy for the best outcome.

The risk-benefit approach to continuing or interrupting therapy for the best outcome is summarized in **Figure 2**.

## **9. Conclusion**

Breast cancer remains one of the most important health problems in the world, both through the physical and mental disability produced among women of all ages. Thus, it is absolutely necessary to find ways to minimize the impact of this disease among the population.

One of the main causes of morbidity and mortality in this group of patients remains cardiovascular diseases due to current oncological therapies, therefore attempting to minimize these undesired consequences by introducing new therapeutic classes, reducing cumulative doses, using new drug associations, and using cardio-protective associations that have shown their preventive role in further degradation of cardiac function has been a priority among cardiologists and oncologists.

A key role, however, is to prevent the installment of cardiac dysfunctions, in addition to preventing further degradation of a dysfunction already established in relation to the oncological therapy or a pre-existing one in a population with cardiovascular risk factors and co-existing cardiac disease. In this regard, it is imperative to carry out a full screening program for the early detection of any functional or morphological impairment of the cardiovascular system among this high-risk population undergoing treatment for breast cancer.

Therefore, more clinical trials on large population groups are required to demonstrate not only the cardio-protective effect of beta-blockers, inhibitors of the renin-angiotensin-aldosterone system such as inhibitors of angiotensin-converting enzyme and blockers of angiotensin receptors used as monotherapy or in therapeutic associations, but also to find the new life-saving therapies that are needed in the management of these patients.

If demonstrating with success their key roles, the urgency becomes implementing an individualized therapeutic regimen based on patient’s comorbidities, type of malignancy and oncology therapy and successfully obtaining the best outcome using this multi-faceted approach.

Moreover, although there are studies that have demonstrated the efficacy of certain classes of drugs in preventing the aggravation of the left ventricular systolic/diastolic dysfunction related to breast cancer therapies, we encourage and support the need for extensive studies over a prolonged period of time to further see and emphasize their effect and to point out their role in prolonging life expectancy in a long-term follow-up program designed for breast cancer survivors.

## **KEY POINTS**

**◊** **Various types of breast cancer treatments (especially anthracyclines and trastuzumab) induce cardiotoxicity even in previously healthy women, not only in those with pre-existing cardiac risk factors**

**◊** **In order to early detect cardiac toxicity, biomarkers assessment and echocardiography can be successfully used**

**◊** **It is crucial to recognize the signs of cardiac dysfunction so that cardioprotective therapy can be initiated**

**◊** **The decision of whether to continue or to interrupt breast cancer treatment is taken by the cardio-oncology team, based on each patient’s characteristics**
